# A Comprehensive Public Health Conceptual Framework and Strategy to Effectively Combat Cholangiocarcinoma in Thailand

**DOI:** 10.1371/journal.pntd.0004293

**Published:** 2016-01-21

**Authors:** Narong Khuntikeo, Watcharin Loilome, Bandit Thinkhamrop, Nittaya Chamadol, Puangrat Yongvanit

**Affiliations:** 1 Department of Surgery, Faculty of Medicine, Khon Kaen University, Khon Kaen, Thailand; 2 Cholangiocarcinoma Screening and Care Program (CASCAP), Khon Kaen University, Khon Kaen, Thailand; 3 Liver Fluke and Cholangiocarcinoma Research Center, Faculty of Medicine, Khon Kaen University, Khon Kaen, Thailand; 4 Department of Biochemistry, Faculty of Medicine, Khon Kaen University, Khon Kaen, Thailand; 5 Department of Epidemiology and Biostatistics, Faculty of Public Health, Khon Kaen University, Khon Kaen, Thailand; 6 Department of Radiology, Faculty of Medicine, Khon Kaen University, Khon Kaen, Thailand; University of Melbourne, AUSTRALIA

## Situation Analysis

Cholangiocarcinoma (CCA) continues to occur with an exceptionally high incidence in the northeast of Thailand, where it causes high levels of morbidity and mortality in the affected individuals and a high socioeconomic burden on their families [1,2]. It has been estimated that there could be as many as 10,000 to 20,000 new cases admitted to surgery each year [3]. Additionally, preliminary data on 47,258 individuals who were screened in the northeast of Thailand showed 42.2% to be infected with *Opisthorchis viverrini* and 2,661 to have CCA [4].

In northeast Thailand, the popular cultural tradition of villagers eating raw or partially cooked freshwater cyprinid fish leads to a high prevalence of infection with the liver fluke *O*. *viverrini* [4,5]. *O*. *viverrini* is classified as a type 1 carcinogen [6] that is associated with the development of CCA. Infection can cause recurrent inflammation, especially of the bile ducts, leading to disorders of the biliary system, including cholangitis, obstructive jaundice, hepatomegaly, fibrosis of the periportal system, cholecystitis, and cholelithiasis [7–10].

Despite taking anthelmintics to treat infections, periductal fibrosis remains. This may trigger malfunctions of the biliary system and is a risk factor for CCA, particularly when associated with such cofactors as nitrite-rich diets, smoking, and alcohol consumption [11,12]. Recently, other potential risk factors for *O*. *viverrini*-associated CCA have been identified, including coinfection with *Helicobacter* [13] and diabetes [14]. In addition, chronic or recurrent infections with *O*. *viverrini* are considered a significant risk factor for developing CCA [6,10,15].

Public health sectors have been campaigning continuously for the prevention and control of liver fluke since 1950, starting with a small, local effort based on education [16,17]. The next stage of the campaign, initiated by the National Health Control Program, included intensive health education and the use of pharmaceuticals (praziquantel) to treat liver fluke infection [14,16,17]. The program was launched in 1984 in four provinces in northeast Thailand and was expanded in 1988 to cover all northeastern provinces. This resulted in a sequential reduction in the average prevalence of liver fluke in Thailand, from 36% in 1988 to 10% in 2002 [16]. This is particularly true for school age children who, 30 years ago, showed a prevalence of up to 95% [18]. These have now dropped to 5.6% in a recent study [19], presumably due to effective education programs. The new, integrated but locally restricted Lawa project [20] aims to combine anthelmintic treatment, intensive health education in communities and schools, and ecosystem monitoring. The value of this approach is debatable due to the costs that arise when extending the program over large areas and the lack of information from villages in which the Lawa program was not carried out, in order to control for natural epidemiological variation.

Nevertheless, the prevalence of liver fluke still reaches high levels in the northeast, although this was found to vary greatly between areas; for example, in Khon Kaen Province it ranged from 2% to 70%, and it was particularly high in the areas close to water sources (up to 40%) [21]. In a sample of 684 individuals from a village in Yasothon Province, who were at least 20 years of age when examined in 2013, 38.6% were infected with *O*. *viverrini* [22]. The total number of opisthorchiasis cases is still estimated to be more than 6 million in the northern and northeastern regions of Thailand [23].

To date, the control programs have all focused only on primary prevention, and none have been extended to secondary and tertiary care. Between 93 and 318 cases of CCA per 100,000 people per year have been reported from Khon Kaen in northeast Thailand [24], with the incidence appearing to increase, not decrease [25,26]. Thus, although the prevalence of opisthorchiasis has, on average, decreased, the incidence continues to be high. This may be due to the lag phase between infection and the development of CCA—as many infections likely occurred before control programs were widespread—or to the recurrent use of praziquantel whenever an infection is diagnosed, although the importance of other factors cannot be excluded [27].

If conventional strategies are implemented that passively tackle the problem without new, proactive approaches, and if health professionals, both at the clinical and fundamental medical research levels, are not given the opportunity to expand their expertise or be equipped with the latest technology, then the rural communities of northeast Thailand will continue to suffer from this fatal disease for an extended period of time. This is because CCA is a cancer that has a slow progression; it is classified under the category of cancers associated with inflammation [28]. Assuming that a community is free from liver fluke today, it will take approximately 20–30 years to successfully achieve an *O*. *viverrini*-associated-CCA-free population because there is no guarantee that those who were infected with the liver fluke in the past will not develop CCA in the future [3].

During its early stages, CCA produces either nonspecific or no symptoms, so that a clinically significant picture only emerges during late stage disease [28,29]. This commonly involves obstructive jaundice, dark urine, pale stools, hepatomegaly, weight loss, abdominal discomfort and, occasionally, high fever with chills [29,30]. As a system to screen the risk group to determine early stage cancer was not available in the past, nearly all of the patients who came to see physicians only did so when they had jaundice or when the cancer was already in its late stage. Currently, surgery is considered the only curative treatment. Curative resection is possible at stages I–III for intrahepatic CCA [31] and stages I–II (and less successfully IIIA and IIIB) for extrahepatic CCA [32]. The earlier the stage at which the disease is diagnosed, the higher the likelihood of a cure with stage 0 cure rates that are frequently high [33,34]; however, in a recent study from northeast Thailand, over 95% of the patients had stage IV disease. In total, 70%–80% of patients received treatment simply to ease obstructive jaundice using a drainage method, or they received various medications (according to their symptoms) because most cases for which staging was determined occurred at the stage IV level [35]. These treatments are merely palliative. Thus, although surgeons play a significant role in the treatment of this disease, their work is currently curative in only 20%–30% of cases [36]. With a screening method that leads to early diagnosis, curative resection would become much more common. How to increase the success of curative surgery is a challenging issue. The proposed solutions for this problem are presented in the following guidelines.

## Cholangiocarcinoma Screening and Care Program (CASCAP) Intervention

### Overview of the intervention

The Khon Kaen University in northeast Thailand—a region with very high incidences of opisthorchiasis and opisthorchiasis-induced cholangiocarcinoma—has created the “Cholangiocarcinoma Screening and Care Program” (CASCAP), in collaboration with the National Health Security Office (NHSO) and the Ministry of Public Health. These organizations have launched a policy to improve the diagnosis and treatment of CCA patients systematically and evenly throughout the northeast of Thailand. This commences with the people who are at risk of developing CCA and those people who already have CCA, so that both groups can receive appropriate treatment from state public health professionals.

The aim of the strategy is to provide long-term screening with follow-up to 150,000 members of the at-risk population (approximately 20 million in Thailand) in order to diagnose precancerous changes to the biliary tract and liver, such as periductal fibrosis and fatty liver, as well as early stage CCA that is accessible by curative treatment [4]. An extensive screening program will achieve this.

Screening will be carried out both by members of the medical faculty in the Departments of Radiology and Surgery at Khon Kaen University, as well as at local primary, secondary, and tertiary hospitals and clinics, after appropriate training by CASCAP members. Subjects falling within the risk group will be actively enrolled from local communities or, if an individual suspects CCA, he or she can apply for an ultrasound examination. At-risk patients with pathology will be referred for additional consultation or a repeat ultrasound after six months; at-risk patients without pathology will be asked to do a repeat ultrasound after 12 months [4]. To date, the CASCAP database has 75,233 examined subjects (October 29, 2015).

This public health intervention will be performed according to the principles of Good Clinical Practice—chapter two of the International Council for Harmonization (ICH) Harmonized Tripartite Guidelines for Good Clinical Practice (GCP), the Declaration of Helsinki, and national laws and regulations about clinical studies. CASCAP was approved by the Khon Kaen University Ethics Committee for Human Research on February 9, 2013 (HE551404).

### Screening, follow-up, and treatment of the at-risk group

At present, a standard method for detecting disorders of the biliary system is the use of ultrasonography [8,37]. Ultrasonographic evaluation is correlated with the pathology of the disease, as well as with the density of *O*. *viverrini* eggs in stools [8,38]. Recently, Chamadol et al. [7] demonstrated that ultrasonography can be used to differentiate the non-tumorous area, with a parenchymal echo pattern, from the cancerous area. This leads to the possibility of early CCA detection. Therefore, an abdominal ultrasound will be a powerful tool to screen and follow up with the CCA risk group in regions where opisthorchiasis is endemic.

This method requires expensive equipment as well as experienced staff. As the effectiveness of diagnosis may be problematic, depending mainly on the skills of the evaluators, it is essential to accelerate the mobilization of radio-diagnostic training to general physicians in public hospitals. Teleradiology is another way to help solve this problem, by substantially expanding the service areas. This requires the purchase of ultrasonography equipment with a satisfactory capacity for the diagnosis and the storage of digital information. It also enables and relies on the establishment and growth of an internet network for consultation by expert radiologists. A preliminary screening system conducted by suitably trained health professionals is an essential prerequisite for program optimization. This can be implemented by determining concrete criteria that are necessary for identifying those people deemed to be at risk of developing CCA. Based on epidemiological data, people with a history of eating raw or partially cooked fish products, who are or have been infected with liver flukes, who have family members dying of liver cancer, or who are 40 years old or older, are most likely to have CCA and fall within the risk group [9,35].

In addition to ultrasound screening, predictive biochemical risk markers for use in detecting patients infected with *O*. *viverrini* or have CCA have an as yet unrealized potential for the rapid screening of people at risk in endemic areas. The most widely studied tumor markers are carcinoembryonic antigen (CEA) and cancer antigen (CA) 19–9. Both CEA and CA 19–9 can be elevated in cholangiocarcinoma [39–41]. However, the sensitivity and specificity, either singly or in combination, are not satisfactory [42,43].

Recently, Yongvanit et al. reviewed the genetic progression and molecular changes in the carcinogenic pathway of liver fluke-associated CCA aimed at assessing patients at risk of developing CCA. This review also discussed the possibility of chemoprevention as a secondary prevention method to reduce the incidence of CCA and summarized [44] the results on altered gene expression, biomolecules, and their modification (for example, DNA adducts, oxidized proteins, oxysterols, and fibrotic markers in hamster- and human-CCA). These multiple-risk biomarkers should now be explored for use in screening for CCA, aimed at chemopreventive intervention for subjects living in endemic areas where the prevalence of opisthorchiasis remains high.

### Networking and training

A network for administering and managing CCA has been developed. This involves the integration of curative and palliative treatment focusing on reducing the rates of mortality and morbidity from surgery, with survival rate and quality of life as the key indicators. There will be four regional hospitals participating in the project: Khon Kaen Regional Hospital, Udon Thani Regional Hospital, Sunprasitthiprasong Hospital, and Maharat Nakhonratchasima Hospital. These super-tertiary hospitals will liaise with the super-tertiary Khon Kaen University Srinagarind Hospital, as the host institution, to implement activities such as the following:

- Developing the capabilities of surgeons, anesthesiologists, nurses handling instruments in operating theaters, nurses in the intensive care unit (ICU), and other relevant professionals involved in curative intent, palliative resection, and bypass.
- Developing procedures for preparing patients for surgery and palliative treatment, covering the following: (1) developing expertise in percutaneous transhepatic biliary drainage (PTBD), (2) developing expertise in the endoscopic implantation of a biliary stent, and (3) developing a method for monitoring and evaluating the therapy provided to the patients afflicted with CCA for curative intent, palliative resection, and bypass.

**Fig 1:**
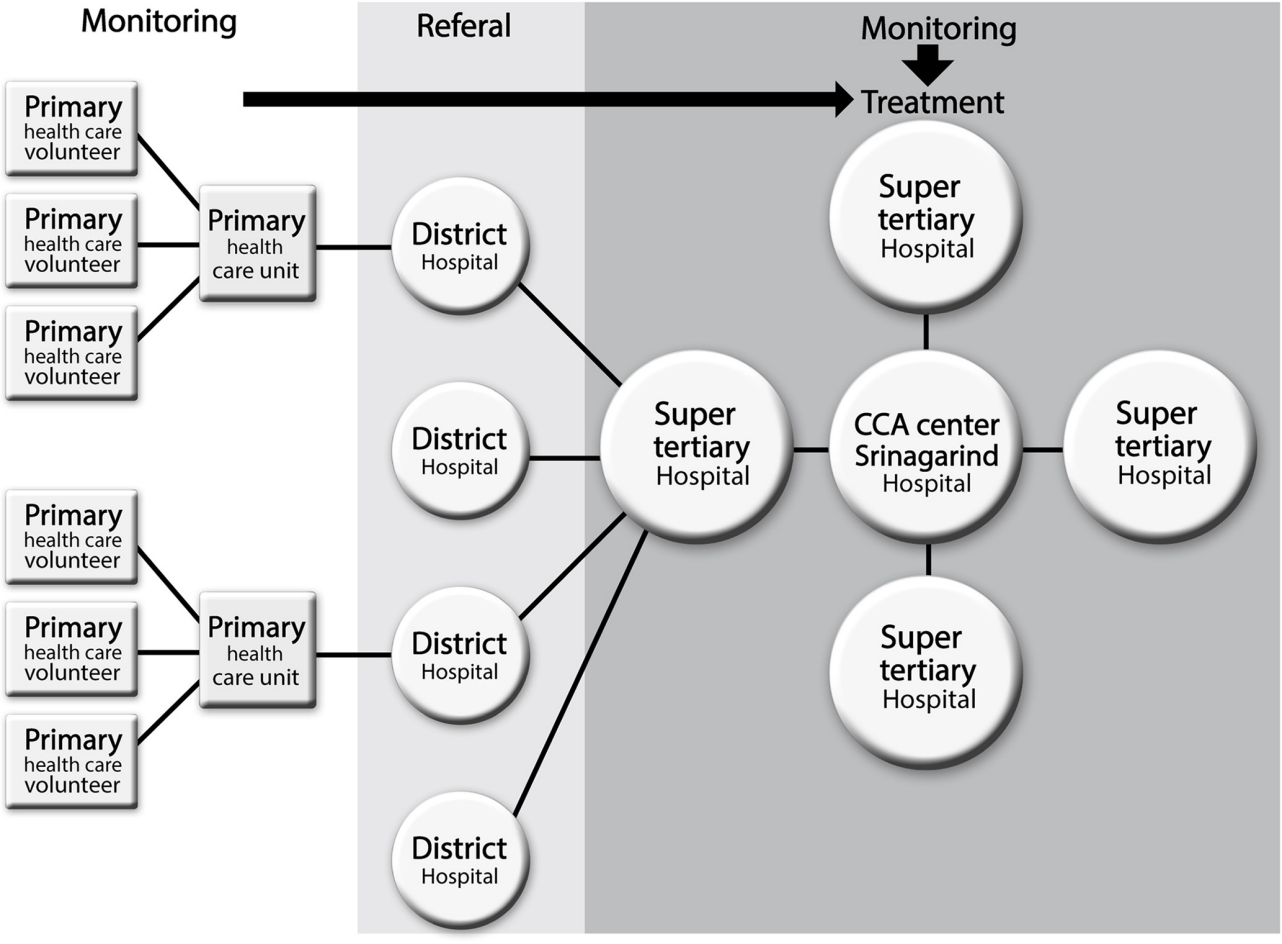
The network hierarchy of cholangiocarcinoma (CCA) screening, therapy, and data management at Srinagarind Hospital, Faculty of Medicine, Khon Kaen University, Thailand as the host institution.

All of these super-tertiary hospitals are of international standard in terms of personnel, diagnostic and clinical equipment, and procedures. All will collaborate to form a network. When this cooperation has been developed, networking will be extended to other hospitals. For example, Udon Thani Regional Hospital will expand the network in the upper northeast of Thailand, Khon Kaen Regional Hospital has an existing network with hospitals in the central northeast, and Sunprasitthiprasong Hospital and Maharat Nakhonratchasima Hospital will build a network with general hospitals in the lower northeast. The network will eventually cover the entire northeast of Thailand. The CCA therapy network hierarchy is detailed in Fig 1.

The following is a list of expected outcomes and dissemination mechanisms for the transfer of information: (1) develop guidelines for screening among the at-risk population; (2) develop guidelines for early diagnosis, pre-operative preparation, curative therapy, and palliative therapy based mainly on the quality of life; (3) establish CCA therapy networks in 20 provinces across the northeast, with the five large hospitals providing core support as hosts; (4) develop a system for effectively monitoring and evaluating the screening and therapy programs of the patients to ensure the continuous development of the service system; (5) develop the ability to increasingly detect more patients at the early stage of CCA development; (6) improve outcomes of treatment of the cases—complete cure, a longer life, and a better quality of life; and (7) achieve the goals of the National Health Policy for CCA management.

As noted above, surgery is the only effective curative treatment for CCA, with major liver resection being necessary for intrahepatic CCA and either major resection or bilio-enteric reconstruction for perihilar CCA. It is thus critical to empower surgeons in all aspects of disease management. This can be done by education, forming the right attitude toward this disease, motivation, and building morale. Training to increase knowledge of new techniques for liver surgery, web-based networking to support both clinical and inspirational aspects, and encouraging young surgeons to actively participate in addressing the burden caused by this problem are all important.

The hospital networking system is hierarchical, with the five super-tertiary hospitals providing the final diagnosis and treatment for patients referred from the primary screening program. The volume of patients arriving, now that the diagnosis of early stage cancer is possible, is increasing so that additional staffs are required and must be trained. Each of the super-tertiary hospitals involved in CASCAP has the capability to train surgeons and nurses in the techniques required for liver resection, post-resection care, and follow-up. This can be made a priority in these institutions.

New surgical guidelines alone are not sufficient to increase the cure rate of this disease. Currently, the diagnosis of cholangiocarcinoma needs to be confirmed by the results of a pathology test. This approach also provides doctors with guidelines for appropriate treatment procedures, namely chemotherapy alone or combined with radiotherapy. In order to extend the joint support line at this point, there must be an increase in the number of trained pathologists available. Additionally, clinical trials should be implemented and accelerated in order to obtain more empirical evidence on the chemotherapy regimens for an improved response against the cancer, at a cost that is feasible for the direct medical reimbursement scheme of the Ministry of Public Health in Thailand. It is widely accepted that for cancer treatment, more than one pharmaceutical product, each related to a different mechanism of action, should be applied, with the aim of achieving synergistic effects. These clinical practice guidelines need to incorporate aspects of pathology, oncology, and radiology. The Thai government recently earmarked 430 million baht (approximately US$12 million) for CASCAP over the next five years.

### Research needs

Because the progress of CCA can be tremendously diverse among patients, in terms of gross- and histopathological-type, and in-depth studies have shown that the activation of genes can also vary greatly, it is difficult to identify a specific tumor marker for CCA. This is unlike certain other cancers, such as hepatocellular carcinoma that has alpha-fetoprotein (AFP) and prostate cancer that has the tumor-specific prostate marker (PSA), to name a couple. Consequently, researchers at the Liver Fluke and Cholangiocarcinoma Research Center of Khon Kaen University are continuing their efforts to discover new tumor markers that are highly effective in assisting with the diagnosis of CCA. This development is based on a variety of scientific methods; for example, the patterns of protein or gene expression in CCA tissues, or the production of monoclonal antibodies that are specific for CCA. High throughput approaches to identify biomarkers for CCA have recently been reviewed [45]. One way that may help to increase their sensitivity and specificity is the use of multi-marker combinations in order to effectively monitor treatment.

As the science of cancer therapy is developing rapidly, it is necessary to conduct targeted therapy research to add more alternative treatments and more effective treatment methods. Targeted cancer therapy is based on the use of certain drugs or substances that can inhibit the growth and spread of cancer cells by interfering with molecular functions that are specific to the growth and spread of these cells [46,47]. When considering the molecular level, targeted cancer therapy is more effective than treatment with other methods, such as chemotherapy or radiotherapy, because it is specific to the cancer cells. The key point is that there are truly small side effects on normal cells [48,49]. At present, studies are being carried out both at the laboratory and clinical trial levels to find new, targeted molecules that are specific for CCA. The results of these experiments demonstrate the feasibility of the use of targeted therapy in the future in the treatment of patients suffering from CCA. Suggested target molecules have been reviewed [50].

One recent advance in this direction has been the development of a new method for detecting *O*. *viverrini* infection by determining species-specific antigens in the urine [51]. The ease and noninvasiveness of urine sample collection and the high diagnostic accuracy of excretory-secretory (ES) antigens from *O*. *viverrini* in urine (urine OV-ES assay) provide an alternative means for the diagnosis of human opisthorchiasis and facilitate the prevention and control of opisthorchiasis. The next step is to initiate the practical application of this method as soon as possible so that primary health care units or district hospitals can detect infected patients and treat them with praziquantel immediately. This should increase the effectiveness of opisthorchiasis control.

Also of critical importance is an understanding of the socioeconomic aspects of CCA at the community level. Which socioeconomic groups are most commonly affected, what burden on the family and community is caused by the search for medical help and through the morbidity and mortality due to CCA, and what measures have been put into place at the community and government levels to alleviate these problems? The huge database collected by CASCAP can help in answering the first two questions and aid in developing policies for the third [4].

Of equal importance is an understanding of the effectiveness of the current control and prevention programs, including education via CASCAP and as part of the regional public health and school-based programs. This information will be needed in order to develop new, integrated measures to control *O*. *viverrini* infection and CCA.

## Conclusions

The elimination of opisthorchiasis and CCA from northeast Thailand, no matter how effective the policy and strategies are, cannot be accomplished effectively without the collaboration of workforces from all sectors, especially the Thailand Ministry of Public Health and NHSO, as the directly responsible bodies. Advanced study centers affiliated with a university as the main coordinating organization will be established to conduct specific research and to pass on knowledge to operational health professionals in the region. A specific requirement is that the public should be well aware of this problem and that they should be willing to cooperate with the medical authorities. Most importantly, it is imperative that willingness, sincerity, and determination to solve this problem are at the forefront of this enterprise. As such, liver fluke infection and cholangiocarcinoma could disappear as major health problems from the northeast and, in due course, from Thailand and neighboring countries.
